# Nitrous oxide induced subacute combined degeneration with longitudinally extensive myelopathy with inverted V-sign on spinal MRI: a case report and literature review

**DOI:** 10.1186/s12883-017-0990-3

**Published:** 2017-12-28

**Authors:** Jun Liang Yuan, Shuang Kun Wang, Tao Jiang, Wen Li Hu

**Affiliations:** 1 Department of Neurology, Beijing Chaoyang Hospital, Capital Medical University, Beijing, 100020 China; 2 Department of Radiology, Beijing Chaoyang Hospital, Capital Medical University, Beijing, 100020 China

**Keywords:** Nitrous oxide, Subacute combined degeneration

## Abstract

**Background:**

Nitrous oxide (N2O), a long-standing anesthetic, is also neurotoxic by interfering with the bioavailability of vitamin B12 if abused. A few case studies have reported the neurological and psychiatric complications of N2O.

**Case presentation:**

Here, we reported a patient of N2O induced subacute combined degeneration (SCD) with longitudinally extensive myelopathy with inverted V-sign exhibiting progressive limb paresthesia and unsteady gait.

**Conclusions:**

This case raises the awareness of an important mechanism of neural toxicity of N2O, and clinical physicians should be well recognized this in the field of substance-related disorders.

## Background

Nitrous oxide (N2O) is a long-standing anesthetic, which also has neurotoxicity by interfering in the bioavailability of vitamin B12 if abused. A few case studies have reported the neurological and psychiatrical complications, even death, related to N2O abuse [1]. Among these complications, N2O-induced myelopathy has been regarded as the most common manifestation [1]. To the best of our knowledge, there are only 18 cases describing N2O-induced subacute combined degeneration (SCD); however, to date, only very rare cases with longitudinally extensive myelopathy with inverted V-sign or “rabbit ears” sign on spinal posterior column. We herein described a 20-year-old female who developed SCD with inverted V-sign on spinal column related to the abuse of N2O.

## Case presentation

A 20-year-old woman presented with progressive paresthesia in her legs and hands, and unsteady gait for 15 days. She had inhaled N2O about 100–200 whipped cream chargers many times daily, for recreational purposes for at least one year. Neuropsychological test showed mild impairment of the cognition, and the Mini mental state examination (MMSE) score was 23. The deficit domains of the MMSE included orientation (minus 3 scores), attention and calculation (minus 4 scores). Neurological examination revealed distal slight weakness, decreased vibration and proprioception, bilateral hyporeflexia, sensory ataxia, positive Babinski sign and Romberg sign.

Laboratory tests revealed decreased level of folic acid (4.40 ng/ml, reference range > 5.4 ng/ml), but the others were normal, including red blood cell, hemoglobin, mean corpuscular volume, serum vitamin B12 (800 pg/ml, reference range 211–911 pg/ml) and homocysteine (8μmmol/L, reference range 0-15 μmol/L). The antibodies of human immunodeficiency virus and neurosyphilis were negative. The results of cerebrospinal fluid test (CSF) were normal for leucocyte count (5/L, reference range 0–8/L), glucose (3.1 mmol/L, reference range 2.5–4.5 mmol/L), and protein concentration (33 mg/dl, reference range 15–45 mg/dl). The inflammatory, immune and infectious biomarkers of both CSF and serum were also unremarkable.

**Fig. 1:**
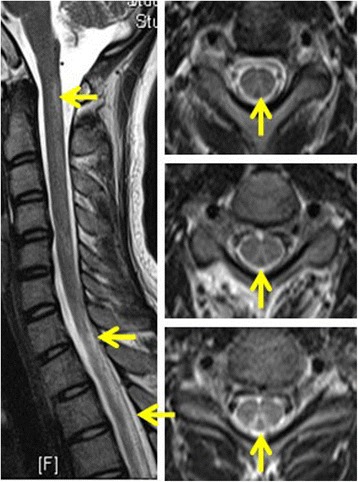
The MRI of spinal cord disclosed abnormal hyperintensities within the dorsal cervical spinal cord. On axial series, V-shaped T2 hyperintensities were again noted within the dorsal cervical spinal cord

**Fig. 2:**
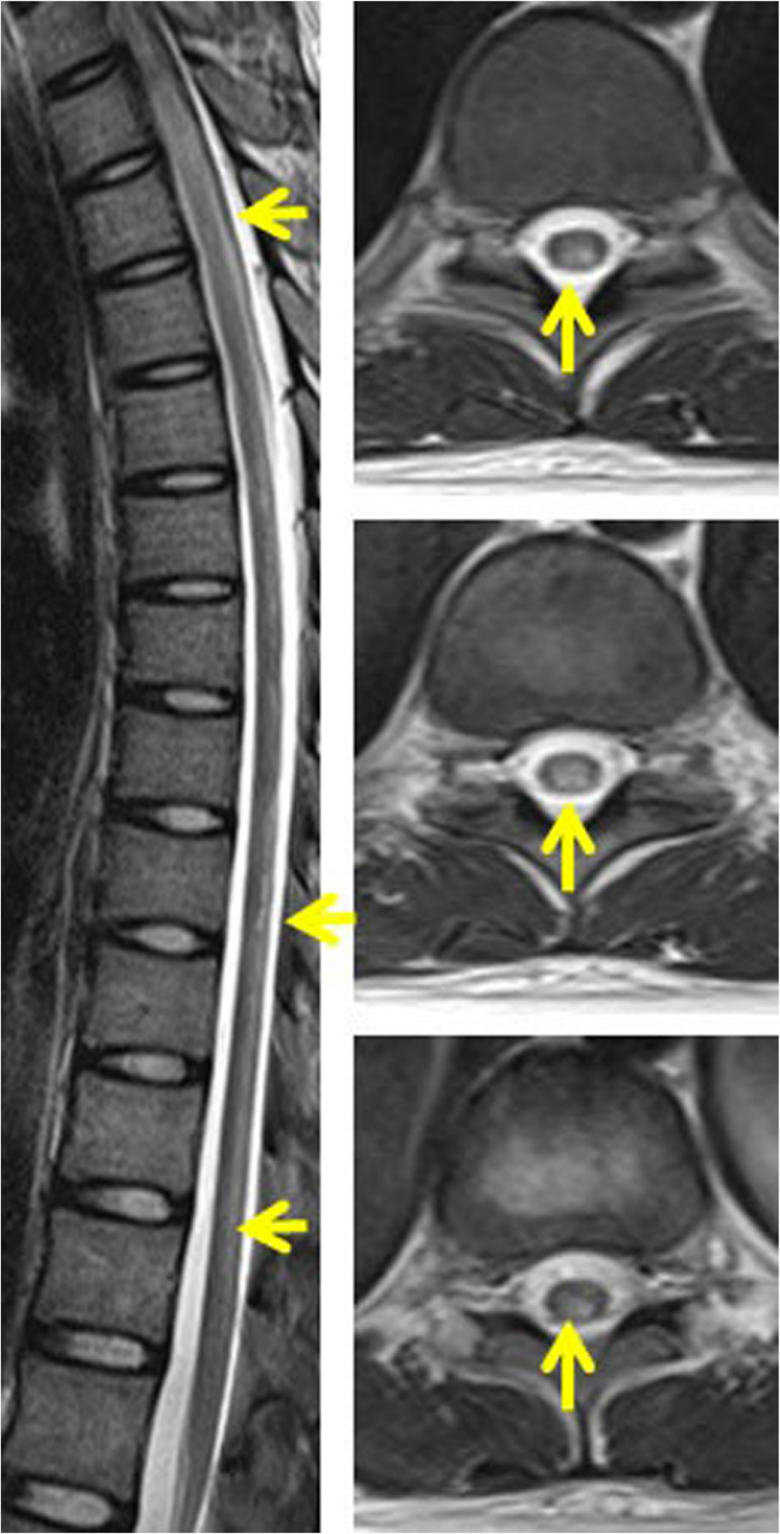
The MRI of spinal cord showed abnormal hyperintensities within the dorsal thoracic spinal cord

The cranial MRI yielded normal findings. The spinal cord MRI showed abnormal longitudinally extensive T2 weighted hyperintensities involving the posterior columns from C1 through T12, with inverted V or “rabbit ears” sign on cervical spinal MRI, but without contrast enhancement (Figs. 1 and 2). Electromyography showed multiple peripheral neurogenic damage, also with decreased nerve conduction velocity and abnormal somatosensory evoked potential. However, visual evoked potential showed normal response.

**Fig. 3:**
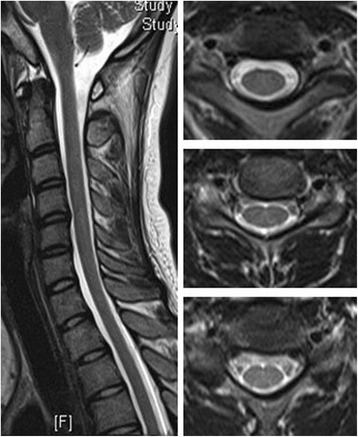
Follow-up cervical spinal cord of MRI revealed the resolution of the previously noted lesions of inverted V-sign within the posterior columns

**Fig. 4:**
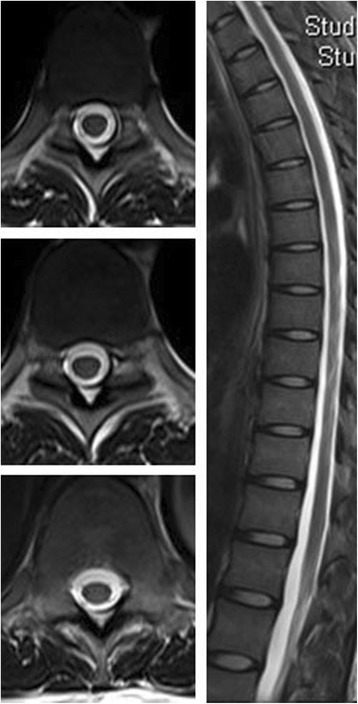
Follow-up thoracic spinal cord of MRI showed significant remission of the posterior columns’ signal alterations

Three months later, with a high dose of supplementation of intramuscular vitamin B12 injections (1 mg per day) and the cessation of N2O exposure, the symptoms of sensation and gait resolved markedly, and the cognitive function fully recovered (MMSE 30). The abnormal hyperintensities of spinal MRI also dissolved with three months’ follow up (Figs. 3 and 4). The diagnosis of N2O induced SCD was supported by clinical history, clinical manifestations, MRI findings, the distinct relationship between N2O exposure, also with the favorable prognosis by the vitamin B12 supplementation.

## Discussion and conclusions

N2O, a well-known anesthetic, has a long history for its recreational use, and its consumption is on the rise rapidly [2]. Several case studies have reported neurological and psychiatric complications of N2O use [1]. To date, there are only 18 cases describing SCD caused by N2O abuse. However, the exact mechanisms of SCD induced by N2O have not been well elucidated. N2O potentially interferes with methionine synthesis by inactivating methylcobalamin [3]. Except for the deficient methylation hypothesis, some other newly discovered functions of B12 in regulating cytokines and growth factors have also been raised [4].

The strengths of our case were listed as follows. Firstly, our case revealed symmetric abnormal signal in the dorsal columns of the cervical and thoracic cord, especially with inverted V-sign on cervical spinal. To the best of our knowledge, only one case has been previously described of such longitudinally extensive myelopathy induced by N2O [5]. Our case also indicated symmetric, reversible changes in the posterior columns correlating well with patients’ clinical symptoms after vitamin B12 supplementation. Secondly, to the best of our knowledge, this is the first report about the coexistence of mild cognitive impairment in patient with SCD by N2O abuse, in spite that the assessment of cognition was only measured by the MMSE. The underlying mechanism of cognitive decline may attributed to the neural toxicity of N2O or the metabolic disturbances from the lower level of metabolites such as folic acid and vitamin B12 or hyperhomocysteinemia [6]. Thirdly, abuse of N2O is common, but generally underestimated especially in developing countries. To date, this is also the first case reported in China (mainland).

In summary, the abuse of N2O has some potentially serious outcomes, especially in young patients presenting with myelopathic symptoms of unclear aetiology [7]. N2O induced SCD may be a very rare manifestation associated with N2O abuse. Early diagnosis and treatment are crucial because it represents a treatable and potentially reversible cause of myelopathy with vitamin B12 [2]. Our case also raises awareness of an important complication of neural toxicity of N2O.

## Abbreviations

CSF: Cerebrospinal fluid test
MMSE: Mini mental state examination
N2O: Nitrous oxide
SCD: Subacute combined degeneration
